# Novel PET Biomarkers to Disentangle Molecular Pathways across Age-Related Neurodegenerative Diseases

**DOI:** 10.3390/cells9122581

**Published:** 2020-12-02

**Authors:** Heather Wilson, Marios Politis, Eugenii A. Rabiner, Lefkos T. Middleton

**Affiliations:** 1 Neurodegeneration Imaging Group, University of Exeter Medical School, London W12 0BZ, UK; h.wilson4@exeter.ac.uk; 2 Invicro, Centre for Imaging Sciences, Hammersmith Hospital, London W12 0NN, UK; ilan.rabiner@invicro.co.uk; 3 Department of Neuroimaging, Institute of Psychiatry, Psychology and Neuroscience, King’s College London, London SE5 8AF, UK; 4 School of Public Health, Imperial College London, London W2 1PG, UK; l.middleton@imperial.ac.uk; 5 Public Health Directorate, Imperial College NHS Healthcare Trust, London W6 8RF, UK

**Keywords:** positron emission tomography, biomarkers, neurodegeneration, precision medicine

## Abstract

There is a need to disentangle the etiological puzzle of age-related neurodegenerative diseases, whose clinical phenotypes arise from known, and as yet unknown, pathways that can act distinctly or in concert. Enhanced sub-phenotyping and the identification of in vivo biomarker-driven signature profiles could improve the stratification of patients into clinical trials and, potentially, help to drive the treatment landscape towards the precision medicine paradigm. The rapidly growing field of neuroimaging offers valuable tools to investigate disease pathophysiology and molecular pathways in humans, with the potential to capture the whole disease course starting from preclinical stages. Positron emission tomography (PET) combines the advantages of a versatile imaging technique with the ability to quantify, to nanomolar sensitivity, molecular targets in vivo. This review will discuss current research and available imaging biomarkers evaluating dysregulation of the main molecular pathways across age-related neurodegenerative diseases. The molecular pathways focused on in this review involve mitochondrial dysfunction and energy dysregulation; neuroinflammation; protein misfolding; aggregation and the concepts of pathobiology, synaptic dysfunction, neurotransmitter dysregulation and dysfunction of the glymphatic system. The use of PET imaging to dissect these molecular pathways and the potential to aid sub-phenotyping will be discussed, with a focus on novel PET biomarkers.

## 1. Introduction

It is widely accepted that age-related neurodegenerative diseases are increasingly becoming a global public health concern—in particular, Alzheimer’s disease (AD) and other late-onset dementias (LOD), with widespread socioeconomic and healthcare impacts worldwide. The increasing burden of age-related diseases is mainly due to the ageing world population and the unprecedented shift in aging demographics of individuals over 60 years of age, which is predicted to rise to two billion in 2050 [1]. Age-related neurodegenerative diseases encompass a spectrum of complex and heterogenous diseases, including AD, Parkinson’s disease (PD), Parkinson’s disease Dementia (PDD), Dementia with Lewy Bodies (DLB), the recently identified dementia form of “Limbic-predominant Age-related TDP-43 Encephalopathy (LATE)”, late-onset forms of Fronto-Temporal Dementia (FTD) and of Amyotrophic Lateral Sclerosis (ALS), as well as parkinsonian plus syndromes, such as Corticobasal Syndrome (CBS), Progressive Supranuclear Palsy (PSP) and Multiple System Atrophy (MSA). Unlike other public health challenges, such as cancer, which have seen the recent development of effective disease modifying treatments, therapies for age-related neurodegenerative diseases remain ineffective to modify the disease course, with most therapies only providing some symptomatic relief.

The majority of research across age-related neurodegenerative diseases is built upon the clinicopathological nosology model [2], whereby a specific clinical phenotype is studied aiming to unlock the underlying pathology, traditionally through post-mortem investigations and, more recently, through in vivo studies, using imaging and other biomarkers that reflect key pathological changes. Variations across diseases have been attributed to the selective vulnerability of specific neuronal subtypes to disease pathology that subsequently determine the clinical phenotypic expression. However, the majority of age-related neurodegenerative diseases are complex in nature, resulting from poorly understood interactions between genomic, environmental and lifestyle factors, across the life course, and harbor multiple pathologies; as a result, their clinical presentations can have distinct, as well as overlapping, features occurring at different levels and timepoints [3,4,5]. The concept that neuronal networks, rather than neuronal subtypes, could underlie differences in the clinical phenotype and the susceptibility of individuals to different neurodegenerative diseases has gained increasing interest over the last decade, aiming to unlock the paradigm of age-related neurodegenerative diseases [4,6,7,8,9]. In 2018, the National Institute on Aging and the Alzheimer’s Association proposed the Research Framework classification of AD to better define the diagnosis of AD, across a disease continuum from preclinical to severe clinical stages from other LOD forms, based on the in vivo AT(N) biomarker signature, corresponding to the three landmark pathological features of increased Amyloid and Tau burden, associated with a significant loss of volume and neurodegeneration [10,11]. While this classification is a step towards the application of biological signature profiles, consideration should be given to the use of a binary classification model for continuous variables, based on a predetermined threshold, as a predictive or diagnostic tool in clinical trials, especially when using a clinical phenotype, such as cognitive decline or dementia, as the primary outcome measure [12].

Almost all age-related neurodegenerative diseases can be classified into sporadic or familial forms. The discovery of fully penetrant genetic mutations in several familial neurodegenerative diseases has allowed for the investigation of the early disease pathology prior to the clinical manifestations, in these familial forms, prior to the manifestation of clinical symptoms that could help to unlock causal pathways. In the nonfamilial sporadic forms, in addition to the genetic variants that are noncausative but can confer susceptibility to disease, there is a wide range of risk factors that may affect disease onset and development, including environmental factors and exposures across the life course, cardiovascular status and hypertension, obesity, diabetes, sleep disorders and a variety of factors related to brain biological aging, such as protein misfolding and aggregation, epigenetics and perturbations in DNA damage and repair. However, not all patients with all, or some, of these risk factors will develop symptoms and signs amounting to a clinical diagnosis within their lifetime. While the interaction between genetics and disease mechanisms is indeed complex and has not been fully elucidated, it has been postulated that unraveling the genetics of age-related neurodegenerative diseases might form the basis for sub-phenotyping and/or reclassification based on genotypic divergence aiming to drive forward the application of precision medicine [13].

**Figure 1:**
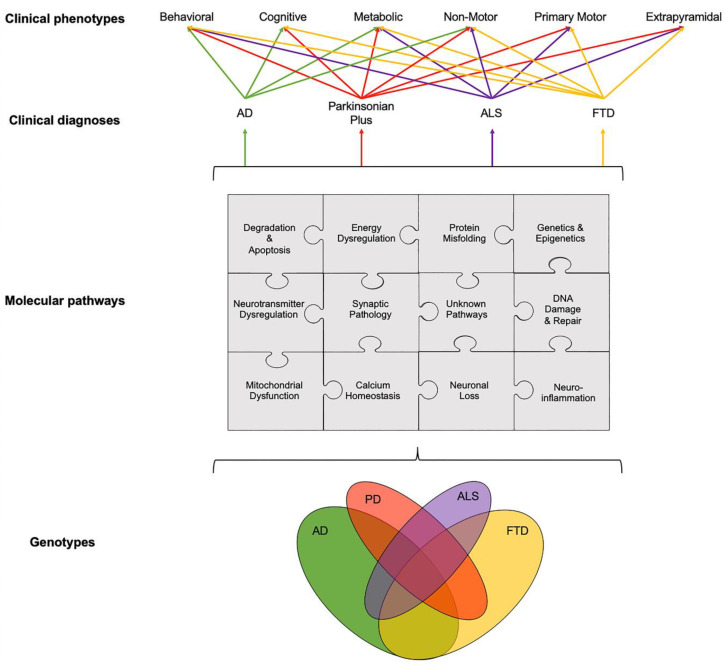
Schematic illustration of interlinked genotypes, molecular pathways and clinical phenotypes across age-related neurodegenerative diseases showing the overlap between various components and pathways at different levels, from genetics and molecular pathways to clinical phenotypes. Disentangling this etiological puzzle of known and yet unknown pathways acting distinctly or in concert could improve the stratification of patients into clinical trials and, potentially, help to drive the treatment landscape towards the precision medicine paradigm. The relationship between clinical diagnosis and clinical phenotypes was adapted from Ahmed et al., 2016 [3]. Abbreviations: AD: Alzheimer’s disease, ALS: Amyotrophic Lateral Sclerosis, FTD: Fronto-Temporal Dementia and PD: Parkinson’s disease.

There is a need to disentangle the etiological puzzle of age-related neurodegenerative diseases, whose clinical phenotypes arise from known, and as yet unknown, pathways that can act distinctly or in concert (Figure 1). Over the last 40 years, preclinical animal studies and post-mortem evaluations have unlocked a number of disease mechanisms and therapeutic targets, which showed promise to translate into novel therapies for age-related neurodegenerative diseases. In AD, the main causal hypotheses involved the amyloid cascade and the tau phosphorylation-propagation hypothesis. However, the majority of clinical trials targeting these mechanisms have failed to meet their primary endpoints [14,15,16]. The failure of clinical trials, across age-related neurodegenerative diseases, could be due to a number of reasons such as the late initiation of treatments in the disease course, poor target engagement or selection of the tested compound, suboptimal cohort stratification and the inability to reach the required effect sizes due to inadequate sample size and/or short follow-up periods [15]. Moreover, an inadequate appreciation of the complexity of disease etiology and pathophysiology can lead to an oversimplified mono-therapeutic approach [16]. 

While preclinical and post-mortem studies have, and will likely continue to, play a key role in the drug discovery process, as well as in understanding the underlying molecular mechanisms, considerations have to be given to their direct translation into humans [14,17]. For example, animal models of late-onset neurodegenerative diseases typically develop symptoms and die young, whereas in humans these diseases typically occur in late life [18]. Furthermore, post-mortem studies provide insights into pathological changes at a single timepoint (the very end stage of the disease), which can be contaminated from chronic drug treatments and other pathologies, making it difficult to disentangle whether the changes observed are a cause or consequence of neuronal death. The rapidly growing field of neuroimaging offers valuable tools to investigate disease pathophysiology and molecular pathways in vivo in humans, with the potential to capture the whole disease course. Positron emission tomography (PET) imaging combines the advantages of a versatile imaging technique with the ability to quantify, to nanomolar sensitivity, molecular targets, both in animals and in living humans. Magnetic resonance imaging (MRI) techniques can offer high spatial resolution and anatomical granularity with advanced acquisition protocols and analysis methodologies offering a platform to explore microstructural and functional connectivity, iron deposition, neuromelanin levels and neuro-hydrodynamics. Therefore, PET and MRI techniques are commonly employed in unison to extrapolate meaningful outcome measures reflecting molecular biology in vivo.

This review will discuss the dysregulation of the main molecular pathways, pathology and biological networks, highlighting where these are distinct and overlapping, across the spectrum of age-related neurodegenerative diseases. The use of molecular PET imaging to disentangle molecular pathways will be highlighted, focusing on novel biomarkers. Finally, the potential for biomarker-driven epigenomic, biological and clinical signatures to improve disease sub-phenotyping for the stratification of patients into future clinical trials towards the precision medicine icon will be discussed.

## 2. Dysregulation of Interlinked Molecular Pathways across Age-Related Neurodegenerative Diseases

While the temporal onset and the rate of progression can vary, clinical phenotypes, such as behavioral, cognitive, metabolic, nonmotor, primary motor and extrapyramidal, often overlap across different age-related neurodegenerative diseases (Figure 1). For example, patients with FTD can present with extrapyramidal symptoms similar to PD; AD patients can experience nonmotor symptoms such as sleep problems, which overlap with nonmotor symptoms observed in PD and parkinsonian plus syndromes, and patients with ALS can present with behavioral symptoms, such as apathy, which can overlap with FTD, parkinsonism plus syndromes and AD [19,20,21]. The pathogenesis and progression of age-related neurodegenerative diseases likely involves a dynamic interaction between various components and pathways at the genetic and pathological levels (Figure 1). Specific PET radioligands have been developed to target some of these molecular components, enabling the exploration of these pathways in vivo. There are a number of genotypic and molecular pathways that show varying degrees of overlap and crossover at various stages of disease etiology and progression. For example, while the clinical phenotype of three causative genes for FTD, *C9orf72, MAPT* and *GRN*, are associated with a similar behavioral variant FTD (bvFTD) presentation, the underlying protein pathology varies such that *MAPT* mutations are associated with tau pathology and *C9orf72* and *GRN* mutations are associated with Tar-DNA-binding protein (TDP)-43 pathology [3]. Furthermore, a number of studies have unlocked genetic signatures that are common across different age-related neurodegenerative diseases. A meta-analysis of 1270 post-mortem brain tissue samples from AD, PD, ALS and Huntington’s disease (HD) patients identified shared gene expression signatures for 243 genes [22]. The common genes identified across these different diseases were related to functional pathways, including inflammation, synaptic signaling, metabolic dysfunction and oxidative stress. Moreover, while the causal role of epigenetics on age-related neurodegenerative diseases remains a topic of debate [23], similarities in the dysregulation of transcriptional networks and protein interaction networks have been reported [5].

**Figure 2:**
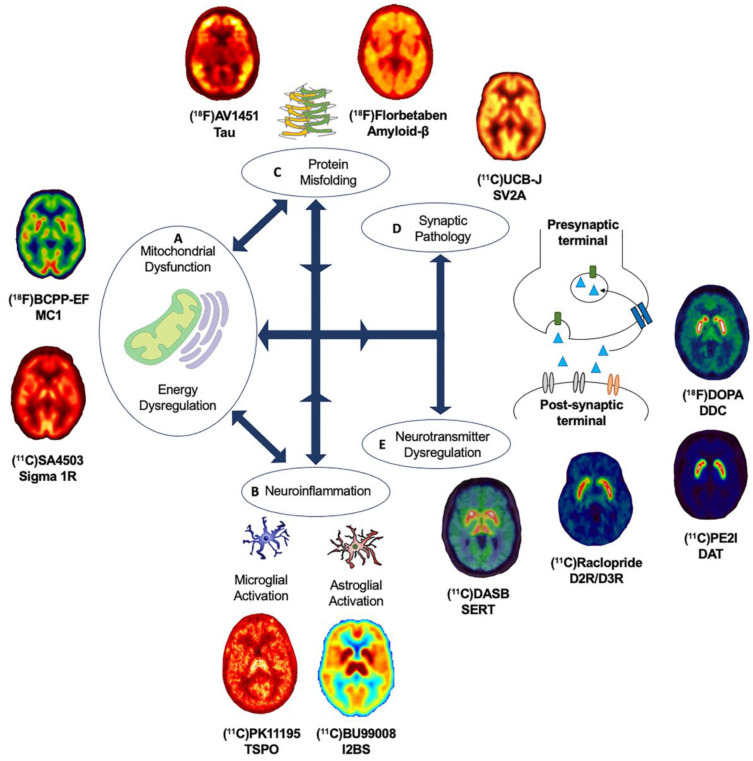
Overview of molecular pathways targeted with PET radioligands. (**A**) Mitochondrial dysfunction and energy dysregulation can be investigated using (^18^F)BCPP-EF, for mitochondrial complex 1 and (^11^C)SA4503 for sigma 1 receptor. (**B**) Neuroinflammation can be investigated by targeting translator protein expressed on activated microglia using PET radioligands such as (^11^C)PK11195 and astroglia activation using novel PET radioligands such as (^11^C)BU99008 for imidazoline 2-binding sites. (**C**) Abnormal protein aggregation of tau and amyloid-β can be quantified using specific radioligands such as (^18^F)AV1451 and (^18^F)Florbetaben, respectively. (**D**) Synaptic pathology can be investigated using (^11^C)UCB-J targeting synaptic vesicle glycoprotein 2A. (**E**) Dysregulation of neurotransmitter systems can be investigated by employing various PET radioligands, including serotonergic markers such as (^11^C)DASB for the serotonin transporter and dopaminergic markers such as presynaptic markers (^18^F)DOPA for dopamine storage, (^11^C)PE2I for dopamine transporter and (^11^C)Raclopride for postsynaptic dopaminergic receptors, as well as PET radioligands for noradrenergic, glutamatergic and GABAergic systems. Abbreviations: D2R/D3R: Dopamine type-2/type-3 receptor, DAT: Dopamine transporter, DDC: Dopa Decarboxylase, I2BS: Imidazoline 2-binding sites, MC1: Mitochondrial Complex 1, SERT: Serotonin transporter, Sigma 1R: Sigma 1 receptor, SV2A: Synaptic vesicle glycoprotein 2A and TSPO: Translocator protein.

It remains to be elucidated why, and how, pathologies diverge towards different clinical phenotypes and if there is a common causal mechanism that links the spectrum of age-related neurodegenerative diseases. The molecular nexopathies paradigm, introduced by Warren and colleagues, proposes that specific pathogenic proteins result in the disintegration of specific neural networks and multiple functional networks, which could give rise to phenotypic variations, as well as overlap between neurodegenerative diseases [4]. A deeper understanding of interlinked and distinctive molecular pathways, which drive pathological and clinical consequences, could provide novel therapeutic strategies. This section will highlight known overlapping, and distinct, molecular pathologies and pathways, focusing on the use of molecular PET imaging, in age-related neurodegenerative diseases (Figure 2).

## 3. Mitochondrial Dysfunction and Energy Dysregulation

There is an increasing body of literature implicating dysfunction of mitochondria and endoplasmic reticulum (ER) dynamics, energy metabolism and oxidative stress within the molecular paradigm of age-related neurodegenerative diseases [24,25,26,27,28,29]. Protein aggregation and deposition have been linked with mitochondrial dysfunction, disrupted mitochondrial transport, dysregulation of adenosine triphosphate (ATP) production, calcium imbalance and oxidative stress [28]. Furthermore, mitochondrial dysfunction can alter the energy supply to synapses, which could drive synaptic disconnection, contributing towards synaptic dysfunction and loss [30,31]. The identification of several genes, such as *PINK-1*, *Parkin, TREM2, APOE* and *TOMM40* [32,33,34,35], which play key roles in the normal functioning of mitochondria has also highlighted the role of mitochondrial dysfunction in disease pathogenesis [36,37,38]. The temporal sequence of events and the exact interplay between mitochondria and ER dysfunction, oxidative stress, neuroinflammation and protein deposition remains to be fully elucidated. There are lines of evidence to support the accumulation of toxic proteins preceding and triggering mitochondrial and ER dysfunction [39,40,41]. Conversely, other evidence suggests that mitochondrial dysfunction and, consequently, oxidative stress and calcium imbalance, together with dysfunction of the ER, may lead to protein misfolding and the accumulation of toxic protein aggregates [42,43]. 

The development of novel PET radioligands, (^18^F)BCPP-EF, for mitochondrial complex 1 (MC1) and (^11^C)SA-4503 for sigma 1 receptor (σ1R) enables the in vivo investigation of mitochondrial and ER dysfunction (Figure 2A) in late-onset neurodegenerative and other diseases related to aging [44,45]. Sigma-1 receptors are expressed at the mitochondrion-associated ER membrane, where they regulate calcium signaling from the ER to the mitochondrion [46,47,48]. Sigma-1 receptors also display neuromodulator and neuroprotective properties, aiding protein folding and modulating synaptic neurotransmitter functions [46,49,50]. MC1 plays a fundamental role in cellular energy production, acting as the first rate limiting step of oxidative phosphorylation in the electron transport chain in mitochondria, as well as maintaining calcium homeostasis and regulating reactive oxygen species (ROS) levels [51,52]. The altered expression and dysfunction of σ1R and MC1 have been illustrated from post-mortem and preclinical studies in ALS, AD and PD [49,50,53,54,55,56,57]. 

Recently, σ1R and MC1 levels were investigated in a cohort of early de novo PD patients using (^11^C)SA-4503 and (^18^F)BCPP-EF PET, respectively [58]. Lower levels of σ1R and MC1 were observed at the baseline, but there were no significant cross sectional or longitudinal changes at 12-months follow-up. In another small cohort of moderate levodopa-treated PD patients, decreased striatal σ1R levels was reported [59,60], suggesting that the loss of σ1R might be more prominent in moderate-to-advanced disease stages. A combined (^18^F)BCPP-EF and (^11^C)PE2I PET preclinical study demonstrated that the striatal loss of MC1 correlated with the loss of presynaptic nigrostriatal dopaminergic neurons, supporting the interplay and colocalization of mitochondrial and synaptic dysfunction in a PD model [61]. Work is ongoing to investigate the role of σ1R and MC1 in AD, ALS, FTD and HD using (^11^C)SA-4503 and (^18^F)BCPP-EF PET, respectively, as part of the MIND-MAPS program (https://lp.invicro.com/mind-maps), which could help to provide a more comprehensive understanding of the mitochondrial-ER-synaptic complex, across the spectrum of age-related neurodegenerative diseases. Preliminary work suggests decreased MC1 density in AD [62] and FTD patients [63], with the loss of MC1 associated with global cognitive impairment across cohorts of age-related neurodegenerative cohorts [64]. Furthermore, preliminary findings indicate that σ1R density is increased in early AD, suggesting that this may represent a potential cellular response to stress that could subsequently decrease as the disease progresses [62]. Reduced (^18^F)BCPP-EF uptake has also been shown to correlate with increase tau deposition, using (^11^C)PPB3 PET, but not with amyloid-β, using (^11^C)PiB PET, or glucose metabolism, using (^18^F)FDG PET [65]. These preliminary findings could indicate that tau pathology precedes early mitochondria-related energy failure. However, these findings need to be further validated in larger, longitudinal studies. The temporal relationship between mitochondrial dysfunction, energy dysregulation and synaptic neuropathology warrants further investigation, as it may play a key role in the development of several age-related neurodegenerative diseases.

## 4. Immune Activation and Neuroinflammation

Neuroinflammation and alterations in the immune response have been linked with multiple pathological processes associated with age-related neurodegenerative diseases [66]. While there is evidence to implicate the role of the adaptive immune system [67,68], the majority of molecular PET imaging research to-date has focused on microglia and astrocytes as part of the innate immune system [69,70,71]. Stress factors, such as misfolded protein aggregates [72], could disrupt the tightly regulated balance between protective and detrimental effects of glial response. This imbalance could result in microglia and astroglia induced neurotoxicity via ROS, proinflammatory cytokines resulting in chronic neuroinflammation and glutamatergic excitotoxicity [73,74,75,76]. It has recently been reported that the crosstalk between microglia and astroglia activation may also contribute to dysregulation of the immune response following the accumulation of protein aggregates, mitochondrial dysfunction and progressive neuronal damage [77]. In ALS, it has been postulated that the shift in glial cells from neuroprotective to neurotoxic effect could contribute towards disease pathology [78]. The identification of genetic mutations in genes regulating microglial activation, such as *TREM2* and *CD33*, in AD, FTD and PD [79,80,81] further supports the potential of a common causal role of a dysregulated immune response. 

The majority of in vivo PET imaging studies investigating the role of neuroinflammation have focused on the use radioligands targeting the 18-kDa translocator protein (TSPO), expressed in the outer mitochondrial membrane and elevated in activated microglia (Figure 2B). A wide variety of TSPO ligands, such as (^11^C)PK1195, (^11^C)PBR28, (^18^F)DPA-714, (^11^C)DAA1106 and (^11^C)ER176, have been employed [82,83,84]. Increased binding of TSPO PET radioligands, interpreted as microglia activation, has been reported in PD [85,86,87,88], PDD [89], MSA [90], PSP [91], CBS [92], HD [93], AD [94,95], FTD [96,97] and ALS [98,99]. Increased (^11^C)PK1195 uptake has also been associated with higher amyloid-β burden in AD [100] and with reduced glucose metabolism in AD and PDD [101], suggesting an in vivo link between microglia activation, protein aggregation and energy dysregulation. Despite their wide application, there are a number of limitations to take into consideration when interpreting results for TSPO PET radioligands, such as the poor signal-to-noise ratio and high levels of nonspecific binding of the first-generation TSPO radioligands and the sensitivity to single-nucleotide polymorphisms in the TSPO gene for second-generation radioligands [100,102]. Third-generation TSPO radioligands, such as (^11^C)ER176 [82,83], which address some of these limitations warrant applications across age-related neurodegenerative diseases. However, the greatest challenge in the use of TSPO radioligands is not related to their imaging characteristics but the complicated biology underlying TSPO density changes. For example, TSPO radioligands cannot distinguish between different neuroprotective or neurotoxic isoforms of microglia, and more generally, there is an ongoing debate over the exact function and role of TSPO upregulation for the immune response.

The identification of reliable molecular targets for microglia activation beyond TSPO has been the focus for the development of the next generation of PET radioligands assessing neuroinflammatory biomarkers. Emerging PET radioligands that could provide key insights into inflammatory pathways include (^11^C)PS13 and (^11^C)MC1 for cyclooxygenase (COX)-1 and COX-2, respectively, and (^11^C)JN717 and (^11^C)SMW139 for the purinergic receptor P2X7 [103,104]. Recently, increased (^18^F)DPA714 PET in the absence of increased (^11^C)JN717 PET has been reported in ALS [99]. Studies are underway investigating P2X7 receptor in PD patients, using (^11^C)SMW139 PET (EudraCT Number: 2018-000405-23). The development of the PET radioligand (^11^C)BU99008 targeting imidazoline 2-binding sites (I2BS) expressed on activated astrocytes [105] has sparked the in vivo investigation of astroglial activation (Figure 2B) across several age-related neurodegenerative diseases [106,107,108,109]. Recently, astroglial activation, reflecting increased (^11^C)BU99008 binding, has been reported in early PD patients with decreased (^11^C)BU99008 binding, possibly reflecting that loss of astroglia function occurs in moderate-to-advanced PD disease stages and is associated with longer disease duration and higher global disease burden [108]. Preliminary findings show increased (^11^C)BU99008 PET in AD, with the highest levels in amyloid-β-positive AD patients [106,109], suggesting a role of astrogliosis in the pathophysiology of AD. Furthermore, preliminary work suggests astroglia activation is present also in PDD, suggesting a potential role in cognitive impairment in later stages of PD [107].

## 5. Protein Aggregates and the Concept of Pathobiology

A common feature across the spectrum of age-related neurodegenerative diseases is the presence of misfolded proteins. The type of protein pathology, as well as the temporal and spatial distribution, can vary between diseases that led to the early pathology-based classification of neurodegenerative diseases, according to the presence and spread of specific misfolded proteins. However, the presence of overlapping pathologies suggests a complex interplay between protein aggregates with disease etiopathogenesis, progression and clinical phenotypes. For example, in AD brains, α-synuclein has been reported to coexist with amyloid-β in senile plaques and in degenerating neuritis [110], and glial tau, neuronal tau and TDP-43 pathology, predominant pathology associated with FTD, limbic-predominant age-related TDP-43 encephalopathy (LATE) dementia and ALS, can also be present in AD and parkinsonian plus syndromes [3,111]. Furthermore, PET imaging has demonstrated that AD-related pathology, such as tau neurofibrillary tangles and amyloid-β plaques, can also coexist within Lewy bodies in PDD and DLB. In DLB, it has been shown that the pathological interplay between tau, amyloid-β and α-synuclein plays a role in the development of dementia [112,113,114,115,116,117], whilst amyloid-β brain accumulation is not typical in PDD [117,118]. Recently, the presence of *APOE-ε4* and *TOMM40-L* alleles has been shown to be associated with the presence of AD-like pathology in DLB, while similar associations were not identified in PDD [33]. Across age-related neurodegenerative diseases, the chronology of mixed protein pathologies, together with multidimensional interactions between genetic and biological pathways, still remains obscure. 

The last decades has witnessed the development and application of a range of PET radioligands to quantify amyloid-β and tau pathology in vivo (Figure 2C). The use of selective PET radioligands led to an eruption of literature describing the presence of protein pathology, the relevance to clinical phenotypes and the relationship with other molecular, structural and functional imaging markers across age-related neurodegenerative diseases [119,120,121,122]. Given the interplay between protein pathologies, it is likely that the development of novel PET radioligands to quantify Huntingtin, TDP-43 and α-synuclein in vivo is required to fully disentangle the relationship between the co-occurrence of complex protein pathologies and molecular pathways of neurodegeneration. Moreover, the ability to reliably measure the spectrum of protein pathology in the human brain would provide the ability to monitor novel multifaceted therapeutic approaches.

Amyloid PET imaging, using (^11^C)PiB, (^18^F)Florbetaben, (^18^F)Flutemetamol and (^18^F)Florbetapir, has been extensively employed to investigate disease specific patterns across age-related neurodegenerative diseases. Amyloid PET imaging can detect the presence of amyloid-β plaques prior to the onset of AD [123], predict cognitive decline in patients with amnestic mild cognitive impairment (MCI) [124,125] and aid the clinical diagnosis of AD [126]. However, amyloid PET does not appear to correlate strongly with cognitive decline once early AD is established [127]. Moreover, significant amyloid-β burden can also be detected in healthy aging individuals without dementia [128,129], suggesting that the presence of amyloid-β plaques alone may not be sufficient to drive cognitive decline. Increased amyloid PET uptake is associated with cognitive impairment in DLB patients [130], while PDD patients show mixed findings with some studies reporting increased amyloid-β levels associated with cognitive decline [114,116] and other studies reporting no relationship between amyloid-β deposition and cognitive impairment [118,130,131]. Therefore, further studies are warranted to untangle the relationship between amyloid-β and clinical phenotypes, as well as the interplay with co-occurrent protein pathologies in vivo. 

PET studies employing tau ligands, such as (^18^F)Flortaucipir [132], have highlighted the association between tau pathology and cognitive performance across the AD continuum [133,134,135], PSP [136], CBS [137,138] and FTD [139] as well as tau co-pathology in Lewy body diseases [140]. A recent study supported the accuracy of (^18^F)Flortaucipir PET visual reads for predicting the presence of AD-like tau pathology at autopsy, suggesting the potential clinical use of (^18^F)Flortaucipir in the diagnosis of AD [141]. A main limitation of first-generation PET radioligands is the presence of off-target binding to neuromelanin and monoamine oxidase [142,143]. The second generation of tau PET radiotracers, including (^18^F)JNJ64349311 [144], (^18^F)APN-1607 ((^18^F)PM-PBB3) [145], (^18^F)MK-6240 [146,147,148], (^18^F)GTPI [149], (^18^F)PI-2620 [150] and (^18^F)RO-948 [151,152,153], offer promising in vivo tools to aid the differential diagnosis between AD and non-AD tau pathology and aid the development of novel pharmacotherapies. Recent findings from (^18^F)PI-2620 PET in AD and PSP illustrate tracer uptake in regions of known tau pathology, in line with post-mortem autoradiography findings [150,154]. Increased (^18^F)PI-2620 uptake within neocortical regions was associated with cognitive impairment in AD [150] and could aid the differential diagnosis of PSP [154]. Another recent study also illustrated that (^18^F)MK-6240 PET could differentiate between AD and FTD, with patterns of (^18^F)MK-6240 uptake in line with Braak’s histopathological staging of tau pathology in AD and negligible (^18^F)MK-6240 uptake in FTD supporting the specificity of this PET tracer for tau tangle conformations [148].

Multifaceted neuroimaging approaches, combining tau PET, amyloid-β PET or FDG PET (for glucose metabolism) with functional and structural MRI techniques, could potentially help to unlock network-based connectivity disease signatures. For example, spatial covariance mapping has been employed to derive network topography underlying clinical phenotypes such as cognitive impairments [155]. Disease-specific networks could also shed light on the relationship between genotypes, disease progression and clinical phenotypes [156]. Recently, the combination of tau PET with functional MRI highlighted the relationship between the spread of tau pathology and alterations in brain functional connectivity in AD, supporting hypotheses for the trans-neuronal propagation of tau pathology [157]. The spread of tau pathology across synaptic connections, in an activity-dependent manner, could also occur in other age-related neurodegenerative diseases.

The dysfunction of cellular degradation systems, including the autophagy-lysosome and ubiquitin-protease systems [158,159], has been linked with oxidative stress, impaired energy metabolism, synaptic dysfunction [158] and the formation of toxic protein aggregates [160,161,162]. Dysfunction of the glymphatic system and sleep disturbances have also been linked with the accumulation of toxic proteins [163]. Across these pathways, it has been postulated that the removal of toxic abnormal protein aggregates may confer neuroprotection [164,165,166,167]. Both the intrinsic and extrinsic apoptosis signaling cascades have also been implicated to play a role in neuronal death and neurodegeneration. However, the exact mechanism and relationship with upstream pathways remains unclear. For example, in PD, *LRRK2* mutations have been linked to mitochondria-dependent intrinsic apoptosis pathways [168], while *PINK1* and *Parkin* mutations show protective effects against stress-induced cytochrome *c* release [169,170]. Neuroinflammation also plays a role as an extracellular driving factor for the activation of extrinsic apoptosis pathways via oxidative insults or proinflammatory cytokines [72,171,172]. While apoptosis could represent a convergence point for different preceding molecular pathways, therapeutic interventions targeting upstream events, prior to activation of the apoptosis pathways, are likely to be more beneficial. PET imaging tools targeting the degradation and apoptotic pathways are currently lacking. The development of such techniques could help to better elucidate their role within the puzzle of molecular pathways underlying protein aggregation and pathobiology.

## 6. Synaptic Dysfunction

Synaptic dysfunction and the loss of synaptic density has been suggested as a common key feature across age-related neurodegenerative diseases [173,174,175,176,177,178]. Under physiological conditions, α-synuclein [179], tau and amyloid-β [180] play a role in supporting synaptic function. However, the abnormal accumulation of these proteins [180,181], alongside neuroinflammation [182], mitochondrial dysfunction [183] and energy dysregulation [30], has been linked with pathological events, including the loss of synaptic integrity and plasticity [184], as well as neurotransmitter dysfunction [185,186], across several age-related neurodegenerative diseases [187]. Anatomical and physiological synaptic dysfunctions could reflect a shared mechanism across neurodegenerative diseases; however, the upstream molecular components leading to synaptic dysfunction, as well as the downstream consequences, may vary between diseases, depending on the synaptic population affected.

Until recently, it was only possible to study synaptic density in post-mortem brain tissue [188]. However, the development of novel PET tracers targeting the presynaptic vesicle glycoprotein 2A (SV2A), which is critical for Ca^2+^ dependent exocytosis [189], paved the way for the in vivo investigation of presynaptic integrity (Figure 2D) [190]. More recently, (^11^C)UCB-J PET has been employed to study SV2A levels in aging [44], idiopathic PD [58,191,192], PSP and CBS [193], as well as across the cognitive spectrum in AD, from preclinical and MCI to advanced clinical dementia [62,194]. In amyloid-β-positive AD patients, decreased (^11^C)UCB-J binding was observed in the hippocampus, which correlated with episodic memory [194]. Preclinical studies support the relationship between synaptic loss and cognitive decline prior to neuronal loss [177,188,195]. Preliminary work suggests that the presence of tau and amyloid-β pathology is linked with synaptic dysfunction across the cognitive spectrum in AD [196,197]. In early drug-naïve PD patients, SV2A loss has been observed in the caudate, putamen, thalamus, brainstem and dorsal raphe, as well as cortical regions [58], with SV2A loss also reported in the substantia nigra in treated PD patients [191,192] and additional SV2A loss in the red nucleus and locus coeruleus in moderate to advanced PD patients [191]. Reduced SV2A was associated with global disease burden and motor symptom severity in early drug-naïve PD patients [58]. In PSP patients, the loss of SV2A has recently been reported in the medulla, substantia nigra, pallidum, midbrain, pons and caudate nucleus, with greatest SV2A loss in the medulla, hippocampus, amygdala, caudate, insula and thalamus in CBD patients with amyloid-β-negative (^11^C)PIB PET scans [193]. The global loss of SV2A in PSP and CBS patients correlated with the total PSP and CBD rating scale as a measure of disease severity and with global cognitive dysfunction [193]. Preliminary work suggests SV2A loss could also be present in PDD and DLB patients [198], as well as in patients with FTD [63]. Work is ongoing to investigate the role of SV2A in ALS, using (^11^C)UCB-J PET as part of the MIND-MAPS program (https://lp.invicro.com/mind-maps), which could help to provide a comprehensive understanding of the role of SV2A across the spectrum of age-related neurodegenerative diseases. Together, these recent findings provide in vivo evidence to implicate synaptic loss in the pathophysiology of age-related neurodegenerative diseases with potential relevance for clinical phenotypes. Ongoing longitudinal (^11^C)UCB-J PET studies, such as the TRAID study [199] and the MIND-MAPS program, will help to shed light on the role of synaptic dysfunction on disease progression. The relationship of synaptic dysfunction with protein pathology, as well as mitochondrial dysfunction and neuroinflammation, warrants investigation across age-related neurodegenerative disorders to better understand the interplay between these molecular pathways.

Prior to synaptic loss, synaptic function and plasticity is likely to be significantly disrupted. The accumulation of protein aggregates has been linked with dysfunction of synaptic signaling pathways, such as impairing long-term potentiation (LTP) [200,201] and enhancing long-term depression (LTD [202]), through the dysregulation of metabotropic glutamate receptors (mGluR) and AMPA and NMDA receptors [203,204,205]. The aggregation and deposition of toxic proteins can also alter synaptic neurotransmitter release by impairing the dynamics of synaptic vesicle endocytosis, recycling, mobilization and storage [185,206,207]. Alpha-synuclein, tau and amyloid-β have also been linked with the membrane trafficking and function of several neuron-specific transporters, including dopamine transporter (DAT), serotonin transporters (SERT) and norepinephrine transporter (NET), which play a critical role in regulating neurotransmitter reuptake from the synaptic cleft [208,209,210,211], as well as glutamate receptor subunits, which play a role in modulating synaptic transmission [212,213,214]. Furthermore, genetic mutations, such as *LRRK2* mutation in PD, have also been shown to impair the dynamics of synaptic vesicle endocytosis, recycling, mobilization and storage [206,207].

## 7. Neurotransmitter Dysregulation

PET imaging studies have collectively demonstrated disruption of neurotransmitter systems (Figure 2E), illustrating both overlapping and distinctive features across age-related neurodegenerative diseases [215]. Neurotransmitter systems have traditionally been a key target for pharmacological intervention based on accumulated evidence that the dysregulation of neurotransmission is closely linked with clinical phenotypes. Here, we focused on the main neurotransmitter systems, cholinergic, serotonergic, dopaminergic, noradrenergic, glutamatergic and GABAergic, highlighting key findings from PET imaging studies across age-related neurodegenerative diseases and shedding light on potential future directions. It is important to acknowledge the potential role of other neurotransmitter systems, including the histaminergic, adrenergic, opioid and cannabinoid systems, which are not captured within this review.

The cholinergic system is predominately associated with the development of cognitive impairment in AD, PD, parkinsonian plus and FTD [120,216]. (^11^C)PMP PET, targeting presynaptic acetylcholinesterase (AChE [217]), has been shown to be reduced in AD patients [218], as well as in PD, PDD and DLB patients [219,220,221]. Furthermore, (^18^F)2FA PET has revealed hippocampal and cortical loss of the postsynaptic α_4_β_2_ nicotinic AChE (nACh) receptor in AD [222] and subcortical and cortical losses in PD patients associated with cognitive decline and depression [223]. Despite the known loss of AChE within the Nucleus Basalis of Meynert in post-mortem FTD tissue [224], no changes in AChE activity were detected in vivo, using (^18^F)MP4A PET in FTD patients [225]. PET studies in PSP, CBS and MSA patients revealed reduced AChE activity within the pons, thalamus and basal ganglia [225,226]. In PSP and MSA, reduced AChE activity in subcortical regions is greater than reductions observed in PD, showing relevance for the development of gait disturbance [226]. Overlap of the cholinergic hypothesis, reflecting neuronal loss in the Nucleus Basalis of Meynert, associated with cortical cholinergic deficits and cognitive impairments, has also been highlighted across AD, PDD and PD [227,228,229,230]. Therefore, while cholinergic dysfunction is likely present across several age-related neurodegenerative diseases, the degree of deficits, as well as the temporal and spatial presentations, is likely different. Future PET imaging of the vesicular acetylcholine transporters, using (^18^F)FEOBV [231], and of the α7-nACh receptors using the novel tracer (^18^F)ASEM [232,233], could provide further insights into the integrity of cholinergic nerve terminals in vivo.

Looking at the serotonergic system, (^11^C)DASB PET has most widely been employed in PD, showing serotonergic pathology starting in premotor stages before the development of overt motor symptoms [234,235] through to advanced disease stages [236]. Serotonergic dysfunction in PD has been linked with the development of nonmotor clinical phenotypes, including depression [237], sleep diseases [238], fatigue [239], weight changes [240], apathy [241] and visual hallucinations [242], in addition to the development of motor phenotypes of tremor [243], levodopa-induced dyskinesias [244] and graft-induced dyskinesias [245]. Albeit to a lesser extent, the serotonergic system has also been implicated in AD, ALS and FTD [246]. (^18^F)MPPF PET studies showing reduced levels of serotonin type 1A (5-HT_1A_) receptors in the hippocampus of AD patients [247]. The development of PET tracers targeting the 5-HT6 receptor, such as (^18^F)2FNQ1P [248,249], could play an important role to further explore the relationship of this serotonin receptor subtype with dementia. In FTD, the loss of 5-HT_2A_ receptors, measured using (^11^C)MDL100907 PET, has been reported to underlie behavior diseases [250]. A (^11^C)WAY100635 PET study also reported decreased 5-HT1A receptor binding in ALS patients within the frontotemporal and cingulate regions [251,252]. Therefore, serotonergic dysfunction across different diseases could involve different serotonin receptor subtypes. (^11^C)Cimbi-36 PET, a 5-HT_2A_ receptor agonist, could offer a valuable tool to investigate changes in serotonin synaptic levels and, indirectly, to measure serotonin release [253,254]. The serotonergic system also interacts with and regulates multiple other neurotransmitter systems, including the dopaminergic, glutamatergic, GABAergic and noradrenergic systems [255]. Given the complexity of the serotonergic system, its central modulatory influences and links with various clinical phenotypes, it could present a principal orchestrator of disease-related pathology and may, perhaps, hold the potential to significantly affect the disease course.

The dopaminergic system underpins the development of primary motor and extrapyramidal phenotypes in PD, parkinsonian plus, HD and FTD but has also been linked with behavioral and cognitive phenotypes in PD, parkinsonian plus, FTD [215,256] and, to some extent, AD [257,258] and ALS [259]. Several PET tracers are available to measure postsynaptic dopamine receptors, such as (^11^C)Raclopride, (^11^C)PHNO and (^18^F)Fallypride, as well as presynaptic dopamine integrity assessing DAT with (^11^C)PE2I, dopamine storage with (^18^F)DOPA and vesicle monoamine transporter type 1 with (^11^C)DTBZ [256,260]. While presynaptic PET markers show some variations, the overarching findings indicate that presynaptic dopaminergic integrity is impaired across PD, PSP, CBS and FTD, associated mainly with rigidity and bradykinesia motor symptoms [215,261]. Across different familial forms of PD, differing patterns of striatal presynaptic dopaminergic loss have been reported, which could reflect the differential influence of specific genetic mutations on molecular pathways towards the etiopathogenesis and progression of PD [262,263]. D2 receptor binding is relatively preserved in PD, while D2 receptor binding is reduced in PSP, MSA and HD; in the latter, decreased D2 receptor binding can be observed during presymptomatic disease stages [264]. A combined (^18^F)DOPA and (^11^C)MP4A PET study illustrated that PDD and DLB share similar dopaminergic and cholinergic deficit profiles [221]. Presynaptic and postsynaptic dopaminergic dysfunctions have also been linked with cognitive and executive dysfunction in PD [265,266,267,268,269,270,271]. The presence and potential role of dopaminergic dysfunction remains unclear in AD, with a recent report suggesting that dopaminergic system could be linked with the pathophysiology of AD [258], whilst another showed no changes in DAT uptake in early-onset AD patients [272]. Decreased striatal DAT binding was also reported in ALS patients [259], although the role of dopaminergic dysfunction in ALS still remains to be elucidated.

The noradrenergic system has been linked with the regulation of several autonomic functions and behavioral and cognitive phenotypes, such as attention, wakefulness, decision making, memory and depression [273,274,275,276]. While there is growing evidence to implicate noradrenergic dysfunction across age-related neurodegenerative diseases [273,277], in vivo studies are generally lacking due to the absence of suitable radioligands. (^11^C)MRB PET, a selective ligand for NET, has been employed to study noradrenergic synaptic terminals in PD [278,279,280]. The PET radioligand (^11^C)RTI-32, a marker of both DAT and NET, has also been employed in PD, with evidence suggesting that the loss of dopaminergic and noradrenergic innervation within the limbic system may play a role in the development of depression and anxiety in the PD disease course [281]. Future studies are warranted, alongside the development of novel PET radioligands, to deepen our understanding of the role of the noradrenergic system across the spectrum of different age-related neurodegenerative diseases and the direct relevance for specific clinical phenotypes.

Glutamatergic neurotransmission is also known to play a central role in supporting higher cognitive function. Excessive glutamate neurotransmission can promote excitotoxic neuronal death contributing to the glutamate hypothesis in AD and FTD [282,283,284]. Recently, (^18^F)FPEB and (^11^C)ABP688 PET studies demonstrated a reduction of hippocampal mGluR5 in early AD, which was associated with lower episodic memory scores and reduced global cognitive function [285,286]. While the relevance of hippocampal mGluR5 loss in AD needs to be fully elucidated, taken together with findings of decreased hippocampal (^11^C)UCB-J binding in AD [194], the loss of mGluR5 could reflect nonspecific synaptic loss. Conversely, the specific synaptotoxicity of amyloid-β at mGluR sites could influence the spatial distribution of mGlu5R loss [287] and could be related to excitotoxin-induced neurodegeneration [288]. Reductions of (^11^C)ABP688 PET uptake have also been reported in patients with the behavior variant FTD (bvFTD) [289]. PET tracers targeting the other metabotropic glutamate receptor subtypes have also been developed, such as the (^11^C)ITMM and (^18^F)FIMX radioligands, which are specific for mGluR1 [290,291]. Given the reported loss of mGluR1 expression in DLB [292], it could be of interest to investigate the role of mGluR1 across several age-related neurodegenerative diseases utilizing these PET radioligands.

The GABAergic system represents the main inhibitory neurotransmitter system within the brain and plays an important role in regulating oscillatory dynamics for cognitive control and work memory function [293,294]. Impaired GABAergic neurotransmission has been linked with HD, FTD, ALS and parkinsonian plus diseases, including PSP. Decreased (^11^C)Flumazenil PET binding, a marker of GABA-A receptor, has been reported in AD [295], PSP [296] and manifest HD [297,298] and linked with motor and extra-motor cortical changes in sporadic ALS [299,300]. The findings that reduced hippocampal (^11^C)Flumazenil PET-binding correlates with memory performance in early AD are in accordance with post-mortem evidence of reduced hippocampal GABA-A messenger RNA expression [295].

## 8. Dysfunction of the Glymphatic System

The glymphatic system is proposed to remove waste products through the cerebrospinal fluid (CSF) to interstitial fluid (ISF) exchange, along the perivascular pathway, with the help of aquaporin-4 water channels, which are polarized in astrocytic end feet towards the capillary vessel walls [163,301,302]. Glymphatic clearance is most active during sleep when aquaporin-4 water channels expand, allowing the removal of metabolic and protein waste products that accumulate during wakefulness [303]. The potential importance of the glymphatic system in the clearance of waste products, including pathogenic protein aggregates, has sparked growing interest to unravel the role of the glymphatic system and sleep dysfunction in the development and progression of age-related neurodegenerative diseases. It can be hypothesized that dysfunction of the glymphatic system, potentially linked with sleep disturbances, may be a common contributing pathogenic factor to several age-related neurodegenerative diseases and, as such, a potential modulable target for therapeutic intervention. The exact relationship between sleep problems, glymphatic dysregulation and protein accumulation in late-onset neurodegenerative diseases remains to be further elucidated.

Dysfunction of the glymphatic system, in late life, has been shown to contribute to the accumulation of amyloid-β [304,305]. Aquaporin-4 knockout mice showed worse cognitive performance and increased accumulation of amyloid-β in the parenchyma and perivascular space compared to wild-type mice [306]. A post-mortem study revealed reduced perivascular end-feet localization of aquaporin-4 in the brains of AD patients [307]. Therefore, redistribution of aquaporin-4 in AD could lead to the dysfunction of glymphatic clearance, resulting in amyloid-β accumulation. Alternatively, the redistribution of aquaporin-4 could be part of the mechanistic actions underlying glymphatic dysfunction. Recently, genetic variants of aquaporin-4 have been associated with sleep disturbances, amyloid-β burden and clinical conversion from MCI to AD [308,309,310]. While the precise role and interactions of aquaporin-4 with the glymphatic system have not been fully elucidated, these findings further support the hypothesis that dysfunction of the glymphatic system could play a pivotal role in neurodegeneration and disease pathogenesis. The PET radioligand (^11^C)TGN-020 has been developed for the quantification of aquaporin-4 [311]. In humans, the distribution of (^11^C)TGN-020 within the brain is consistent with known distributions of aquaporin-4—namely, subpial and perivascular end-feet of astrocytes and choroid plexus [311]. Therefore, (^11^C)TGN-020 PET could have useful applications to help unravel the molecular mechanism by which aquaporin-4 interacts with the glymphatic system and the relationship with disease pathology in age-related neurodegenerative diseases. 

Glymphatic MRI, with intrathecal injections of MRI-based contrasts acting as a CSF tracer, has been developed to quantify CSF-ISF exchange in vivo [312,313]. Recently, delayed clearance of the CSF tracer in the entorhinal cortex was observed in cognitively impaired patients with idiopathic normal pressure hydrocephalus [314,315], a potentially treatable form of cognitive impairment, in late life [316], which may exhibit AD-like pathological features, including the deposition of amyloid-β and tau [317]. The in vivo findings that patients with cognitive deficits display delayed clearance of the CSF tracer within regions involved with cognitive function further supports the hypothesis that dysfunction of the glymphatic system may contribute to the development of dementia and merits further investigations in the preclinical disease stages. The development of a PET radioligand to assess glymphatic function at a molecular level could help to better delineate the role of glymphatic dysfunction in disease etiopathogenesis and pathophysiology. 

The development of novel glymphatic system MRI methodologies, such as 3D phase-contrast MRI [318,319,320,321,322,323,324,325,326], ultra-fast encephalography MRI [327], near-infrared spectroscopy and ultra-fast functional MRI [328], have gained growing interest to better understand fluid dynamics within the brain and to study the role of neuro-hydrodynamics with respect to the glymphatic system. Arterial spin labeling and diffusion tensor imaging techniques are also being employed to assess CBF crossing the blood-brain interface [329] and the movement of fluid in the perivascular space [330,331], respectively. Seven tesla MRI techniques have been proposed as the optimal imaging modality to successfully measure the perivascular space [332,333,334]. The topographical distribution of high-grade MRI-visible perivascular spaces in patients with vascular dementia, cerebral amyloid angiopathy and AD may be overlapping but may also occur in distinct brain regions [335,336,337]. Furthermore, dilated perivascular spaces in the basal ganglia have been linked with high total tau levels in the CSF, indicating neurodegeneration [335]. Interestingly, it has been reported that the severity of MRI-visible perivascular spaces in the basal ganglia are associated with clinically diagnosed subcortical vascular cognitive impairment and negatively predicted AD, whilst the severity of such lesions in the centrum semi-ovale is associated with clinically diagnosed AD [334]. Such studies provide further in vivo evidence on the role of glymphatic dysfunction in different late-onset dementia types. Mathematical models of the glymphatic system are also being developed to better understand the glymphatic system dynamics and pathways under physiological and pathological conditions, as well as to provide quantitative maps for understanding disease pathophysiology and monitoring disease progression [338,339,340,341,342,343]. The continuous development of noninvasive imaging techniques and computational models will allow for the wider application of future in vivo studies investigating the role of the glymphatic system across the spectrum of age-related neurodegenerative diseases.

Preclinical animal models have shown that the clearance of amyloid-β is dysregulated under conditions of sleep deprivation and with advanced aging [344,345,346,347]. Preliminary in vivo studies support the role of sleep in perivascular clearance [348]. In healthy humans, the amyloid-β burden is increased after one night of sleep deprivation [349]. An (^18^F)FDG PET study revealed a reorganization of regional cerebral metabolic activity following sleep deprivation in healthy individuals, which was associated with the decline in cognitive performance observed after sleep deprivation [350]. PET studies using (^11^C)PiB to measure the brain amyloid-β load have demonstrated that adults who report less adequate sleep, more sleep problems and greater somnolence have a greater amyloid-β burden in AD-sensitive brain regions, independently of the APOE-ε4 genotype [351,352]. Based on the above data, it could be hypothesized that, in older adults, sleep disturbances may lead to an increasing dysfunction of the glymphatic system, which could subsequently contribute to increasing the amyloid-β and tau loads. However, the temporal sequence of sleep problems and disease onset, i.e., whether they represent a contributing pathogenic factor (in the preclinical AD stages) or an early clinical manifestation of MCI due to AD, still remains to be determined [347,353]. PET studies performed in healthy volunteers have also implicated changes within the dopaminergic and serotonergic systems related to sleep deprivation, including downregulation of the D2/D3 receptors in the ventral striatum [354,355,356], with no changes in dopamine release [354] and an upregulation of the 5-HT_2A_ receptors in frontal and parietal cortices [357]. Together, these findings suggest a potential interplay between sleep, glymphatic dysfunction and disease pathology, which merits future investigations across several neurodegenerative diseases associated with aging.

## 9. PET Imaging and Personalized Precision Medicine in Advancing the Precision Medicine Icon to Aid Sub-Phenotyping

Challenging the traditional clinicopathological nosology of individual age-related neurodegenerative diseases may open avenues for dissecting disease entities, such as AD or PD, and unraveling their potential heterogeneity into subtypes through in vivo multifaceted biomarker studies. Each with a distinct biomarker-based signature profile, reflecting its distinct pathogenic pathway(s). This approach is not dissimilar to the stratification paradigm, which has, recently, proven its value in several cancer types. Significant recent advances in cancer biology and a biomarker-based subclassification allowed for targeted precision pharmacological interventions, showing significant therapeutic benefit in evidence-based stratified patients, whereas they had previously failed to meet the desired effects [358]. Genetic-based risk prediction models, alongside imaging biomarkers and known risk factors involving vascular and metabolic comorbidities and lifestyle factors, are currently being evaluated for stratifying aging individuals into AD risk groups [359,360,361,362,363,364,365,366,367,368]. Furthermore, disease-specific network patterns, identified using PET and functional MRI techniques, could prove beneficial to unravel network alterations across diseases and to determine the effectiveness of therapies aiming to modulate underlying disease pathways [156,369]. The in vivo investigation of epigenetics, from the preclinical stages and during disease progression, could unlock new insights into gene regulatory processes, disease pathophysiology and novel targets for therapeutic intervention [370]. Imaging epigenetics may now be possible with the recent development of the (^11^C)Martinostat PET radioligand to measure class I histone deacetylase (HDAC) enzyme density [371]. 

While PET imaging can be complex, expensive and its wider application may be limited due to infrastructural and logistical constraints, PET imaging remains the only reliable noninvasive in vivo solution to quantify molecular targets and pathologies in patients across the disease life course. The continued development and wider application could help to transfer promising PET techniques from a research to a clinical setting. There is a need for more longitudinal studies to answer a number of key neurobiological questions and to determine the use of PET radioligands as reliable biomarkers to track disease progression. Furthermore, future work should focus on understanding the relationship between PET biomarkers and early signs and symptoms of age-related neurodegenerative diseases. Capitalizing on fully penetrant genetic mutations could allow for the investigation and characterization of early disease pathology in preclinical stages and, accompanied with longitudinal follow-ups, could help to shed further light on the relationship between these PET markers and early disease symptomatology. Recent methodological advances to measure amyloid-β and tau loads from PET imaging, with higher sensitivity and lower variability, could further improve the accuracy of imaging phenotypic stratification and increased power to detect meaningful outcomes and biological effects in future clinical trials [372,373]. Novel tools such as Amyloid^IQ^ and Tau^IQ^ may add future value to the recent AT(N) classification of AD [10,11]. The scientific community should strive to deepen and enhance these approaches by capitalizing on the next-generation PET radioligands and novel PET biomarkers (Table 1), combined with advanced MRI and computational methodologies. Advances in the next generation of PET radioligands for neuroinflammation, alongside the development of novel radioligands targeting mitochondrial functions, could help to unravel the interplay between glia-related neuroinflammation, abnormal protein aggregation and mitochondrial dysregulation. In the case of neuroinflammation, there is a need to better understand how TSPO changes relate to underlying pathology and to further explore novel molecular targets beyond TSPO, such as cyclooxygenase, purinergic receptors and astrocytes. The development of a PET radioligand specific for α-synuclein, Huntingtin and TDP-43 would be ground-breaking and result in significant advances within the field, including aiding diagnostics; tracking disease progression and the ability to monitor and assess novel drugs aiming to reduce α-synuclein, Huntingtin or TDP-43 levels. Furthermore, the development of such PET radioligands could aid the stratification of patients into future clinical trials and, potentially, help to drive the treatment landscape towards the precision medicine paradigm.

## 10. Conclusions

The scientific community is slowly moving away from the traditional clinicopathological disease models towards a precision medicine paradigm, which is still lacking in age-related neurodegenerative diseases. Based on the available understanding of interlinked genomic, biological and clinical pathways across diseases, it is likely that a dynamic multifaceted biomarker approach is required to disentangle mechanisms of disease pathogenesis and clinical trajectories. This might include markers of pathology spanning from genetic, epigenetic, proteomic, metabolomic and transcriptomic, clinical and digital and molecular PET and MRI imaging to biological CSF and blood-based biomarkers. Unraveling disease-specific network changes underpinned by genetic and pathological patterns could aid the advancement of personalized precision medicine by providing novel diagnostic tools and stratification approaches, as well as in identifying new therapeutic targets. Molecular PET imaging will likely continue to offer an invaluable tool, reflecting molecular pathology, contributing towards unlocking disease mechanisms and identifying biomarkers throughout the disease course. It is hoped that advances in the development of novel PET radioligands (Table 1) and their applications in age-related neurodegenerative diseases, as well as in healthy aging, will help to drive advancements in sub-phenotyping to aid precision medicine-based approaches in diagnosis, prevention and clinical management.

## Figures and Tables

**Table 1:** Overview of new PET radioligand for novel targets and related molecular pathways across age-related neurodegenerative diseases.

PET Radioligand	Target	Molecular Pathway
**Protein aggregation**		
(^18^F)PI-2620 (^18^F)MK-2640 (^18^F)RO-948 (^18^F)GTPI (^18^F)JNJ64349311 (^18^F)APN-1607	Tau	Tau deposition
**Neurotransmitter Dysregulation**		
(^11^C)Cimbi-36	5-HT_2_R	Serotonergic system
(^18^F)2FNQ1P	5-HT_6_R	
(^18^F)FEOBV (^18^F)VAT	VAChT	Cholinergic system
(^18^F)ASEM	α7-nAChR	
(^11^C)ABP688 (^11^C)AZD9272 (^11^C)FPEB	mGluR5	Glutamatergic system
(^11^C)ITMM (^18^F)FIMX	mGluR1	
(^18^F)ΜΝΙ-444	A2A	Adrenergic system
(^11^C)MK-8278 (^11^C)GSK189254	H_3_R	Histaminergic system
**Neuroinflammation**		
(^18^F)DPA-714 (^11^C)ER176	TSPO	Microglial activation
(^11^C)BU99008	I_2_BS	Astroglial activation
(^11^C)JNJ717 (^11^C)SMW139 (^18^F)JNJ-64413739 (^11^C)GSK1482160	P2X7	Purinoceptors
(^11^C)PS13	COX-1	Cyclooxygenase
(^11^C)MC1	COX-2	
**Synaptic Pathology**		
(^11^C)UCB-J (^18^F)UCB-H	SV2A	Synaptic function
**Mitochondrial dysfunction and energy dysregulation**		
(^18^F)BCPP-EF	MC1	Mitochondrial function
(^11^C)SA-4503	σ-1R	Mitochondrial-associated membrane
**Epigenetics**		
(^11^C)Martinostat	HDAC	Epigenetics
